# Energy Options for Wireless Sensor Nodes

**DOI:** 10.3390/s8128037

**Published:** 2008-12-08

**Authors:** Chris Knight, Joshua Davidson, Sam Behrens

**Affiliations:** 1 CSIRO Energy Technology, PO Box 330, Newcastle NSW 2300, Australia. E-Mail: sam.behrens@csiro.au; 2 School of Maths, Physics & Information Technology, James Cook University, Townsville QLD 4811, Australia. E-Mail: joshua.davidson@jcu.edu.au

**Keywords:** Energy Harvesting, Energy Storage, Wireless Sensor Networks, Sensor Nodes

## Abstract

Reduction in size and power consumption of consumer electronics has opened up many opportunities for low power wireless sensor networks. One of the major challenges is in supporting battery operated devices as the number of nodes in a network grows. The two main alternatives are to utilize higher energy density sources of stored energy, or to generate power at the node from local forms of energy. This paper reviews the state-of-the art technology in the field of both energy storage and energy harvesting for sensor nodes. The options discussed for energy storage include batteries, capacitors, fuel cells, heat engines and betavoltaic systems. The field of energy harvesting is discussed with reference to photovoltaics, temperature gradients, fluid flow, pressure variations and vibration harvesting.

## 1. Introduction

Reduction in size and power consumption of consumer electronics has opened up many new opportunities for low power wireless sensor networks. Such networks have significant potential in a variety of applications, including monitoring of animal health and behaviour, structural monitoring for mining equipment and measuring water salinity levels of oceans and rivers.

With these opportunities come a number of new challenges. Sensor nodes are usually battery powered, so as sensor networks increase in number and size, replacement of depleted batteries becomes time consuming and wasteful. Additionally, a battery that is large enough to last the life, say five years, of a sensor node would dominate the overall size of the node, and thus would not be very attractive or practical. Additionally, the battery chemistries often involve toxic heavy metals, and present disposal issues, regardless of rechargeable technology.

As a result, there is a clear need to explore novel alternatives to power sensor networks/nodes, as existing battery technology hinders the widespread deployment of these networks. By harvesting energy from their local environment, sensor networks can achieve much greater run-times, years not months, with potentially lower cost and weight.

Power for wireless sensor nodes can be split into two main technology categories: energy storage and energy harvesting. This paper reviews the state-of-the art technology in each of these fields, outlining different powering options for sensor nodes. These include energy storage utilizing batteries, capacitors, fuel cells, heat engines and betavoltaic systems and energy harvesting methodologies including photovoltaics, temperature gradients, fluid flow, pressure variations and motion harvesting.

Energy storage is the basis of present technology and involves powering the sensor node from energy stored at the node; a key example of this is batteries. This energy may be stored in different forms ranging from electrical charge to hydrocarbon based fuels. By itself, energy storage cannot deliver energy indefinitely, as at some stage the energy will be depleted and need replenishing. The metric used for comparison of these devices is their average energy density, Joules per unit volume; typically this is J/cm^3^.

Energy harvesting is a newer approach and relies on technology to gather energy from the surrounding environment using, for example, solar cells or fluid turbines. It involves converting the ambient energy inherent in the sensor node's environment into electrical energy. By doing so, a sensor node will have the opportunity to extend its life to a range determined by the failure of its own components rather than by its previously limited power supply. The metric used for comparison of energy harvesting devices differs from that used for energy storage as they don't have a fixed amount of energy intrinsic to their volume. Therefore, energy harvesting devices will be rated on their average power density or Watts per unit volume, W/cm^3^, rather than their average energy density.

In general, energy harvesting will not directly power a sensor. This may be because the levels of power are too low, or it may be as a result of the power being in the wrong form. Typically, sensors and nodes require a voltage in the range 2 – 10 V and peak *direct current* of approximately 100 mA. Some energy harvesting techniques generate much higher voltages, produce AC power, or simply do not have sufficient power to run the node directly. The result of this is that electronics are required to condition the power for the device and, critically, secondary energy storage in the form of capacitors or rechargeable batteries will be required. Section 4 deals with issues of power conditioning.

Many of the power options involve taking a technology which has been proven on large scale applications and scaling it down to dimensions suitable for the sensor node. This approach often runs into technical difficulties due to different effects which come into play at smaller scales. Some of these effects, which are detailed in this report, include thermal effects as a device's ratio of surface area to volume changes, viscosity issues involving fluid flow at smaller scale and problems related to increasing volume taken up by battery connectors, packaging and other essential hardware. However, through the persistent work of researchers, many technologies have overcome these obstacles and are nearing fruition.

It should be noted that in the context of this review, the term micro-scale is used to describe nodal elements with sizes of approximately 100 mm on a side, masses of less than 100 gm (not including batteries or associated transducers) and power requirements of less than 100 mW.

To give examples of the energy requirements of sensor nodes, Table 1 shows a number of commercially available nodes and their various levels of power consumption.

The Fleck3 is a CSIRO product and a range of data was easily sourced, unlike the XBeeTM and MICAzTM which are both commercial products, for which full specifications were unavailable. The power consumption is very dependant on the various transmit/store duty cycle components and should not be interpreted as a measure of the efficiency of the device. Note that the nodes in Table 1 are often able to operate at lower power consumption. It is not the purpose of this review to compare individual nodes and manufacturers. Rather, the purpose of Table 1 is to indicate the relative amount of energy required for each node, for the following arbitrary duty cycle: In every 3 minute cycle, 1% (1.8 s) listening, 20 ms transmit time, and the remainder (178.18 s) sleeping.

The final two columns in Table 1 show the number of alkaline AA cells needed to power the node and the length of time they will last for the given duty cycle. Alkaline AA cells were chosen as alkaline chemistry is well established, has a reasonable shelf life, AA cells offer a good trade-off between capacity and size (2850 mAh from 8.3 cm^3^ [2]) and that most readers would have some familiarity with them as they are commonly used in household devices. Note that the values quoted in Table 1 do not allow for powering sensors connected to the device. Typically, the power requirements of some sort of physical sensor to measure temperature or humidity, for example, need to be considered. The magnitude of this power could easily exceed the power requirement of the node itself, effectively halving the battery life.

## 2. Energy Storage

### 2.1. Batteries

The most common power sources for wireless sensor nodes are batteries. Batteries combine good energy density with a range of commercially available sizes while also supplying their energy at precisely the voltage levels required of modern electronics, eliminating the need for intermediate power conditioning electronics. A battery can store energy chemically and can release it as electricity through a chemical reaction which transfers electrons from its anode to its cathode. The power output of a particular battery is limited by a number of factors including: the relative potentials of the anode and cathode materials, and the surface area of the electrodes.

Batteries can be classed in two main categories, primary and secondary. Primary batteries are not easily recharged using electricity, while secondary batteries can reverse the chemical reaction through a recharging process whereby energy is delivered back into the battery and stored in the form of chemical bonds.

When using primary batteries the lifetime of the sensor node is determined by the fixed amount of energy initially stored in the battery. The amount of energy stored depends on the energy density and volume of the battery. For sensor node applications, it is desirable to minimise the volume, and with improvements in battery energy density reaching a plateau, batteries are forcing a large trade-off between the node's lifetime and its volume.

The capacity of a battery is specified by the manufacturer and is achieved by the use of specific discharge rates. Each manufacturer can use their nominated methodology, of which there are many. As an example based on [2], the following is used for non-rechargeable batteries: A discharge rate of 25 mA is applied until the voltage reaches 0.8 V. The time in hours that is taken is then multiplied by the discharge rate (25 mA) to calculate a milliamp-hour (mAh) capacity. An alkaline battery that cannot be recharged has effectively reached the end of its life at 0.8 V. It still contains significant ‘overhead’ energy but this energy is unable to be used. When the battery is thrown out this energy is effectively wasted. This does not apply to rechargeable batteries, as they can be topped up hundreds of times.

For rechargeable batteries, a similar methodology is nominated by a manufacturer to achieve the rated capacity. For example [2] bases their capacities on a 0.1 capacity charge followed by a 0.2 capacity discharge. So a rechargeable battery with a stated capacity of 1,000 mAh will only get that capacity if it is charged at a maximum of 100 mA (for 10 hrs) and then discharged at 200 mA (for 5 hrs). Other examples of discharge rates include a 1 hour rate and a 20 hour rate. Thus a 1,000 mAh battery will achieve this capacity using the 1 hour rate if discharged at 1,000 mA. The 20 hour rate is typically used on sealed lead acid batteries as they do not perform well at the 1 hour rate. Importantly, a battery will only achieve the nominated capacity if discharged at the nominated rate. A higher or lower discharge rate will result in a different capacity due to internal energy changes. Typically if a lower discharge rate is used then the battery will supply a slightly higher capacity. This is important for wireless nodes as they typically consume much less than the nominated discharge rate, and thus the battery should last slightly longer than predicted from the capacity. At very low discharge rates shelf life becomes an issue which is addressed below.

**Figure 1.**
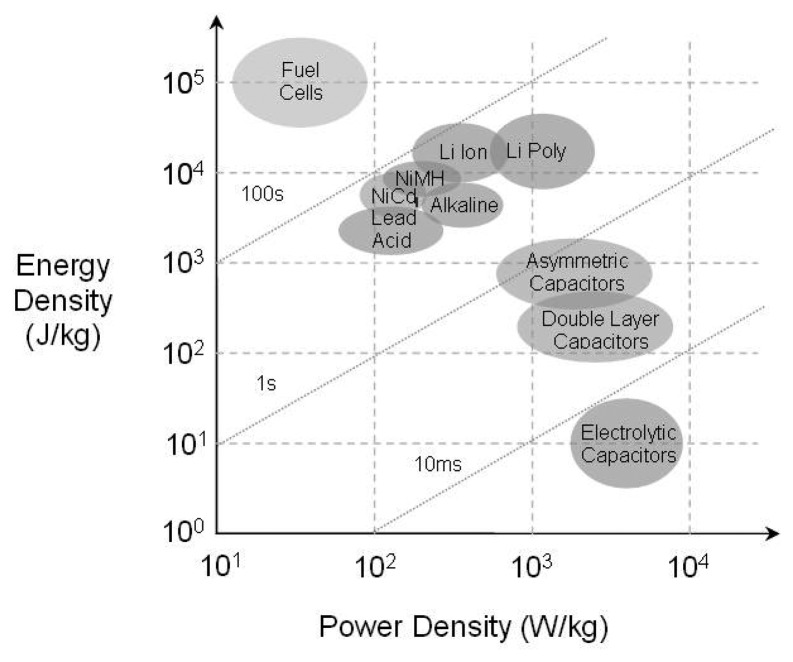
Ragone chart for capacitors, supercapacitors, batteries and fuel cells.

Like primary batteries there are different types of secondary batteries whose characteristics are determined by their internal chemistries. Conventional chemistries such as Nickel-Zinc (NiZn), Nickel Metal Hydride (NiMH) and Nickel-Cadmium (NiCd), offer high energy densities and good discharge rates, but with the disadvantages of short cycle life and adverse “memory” effects. Lithium-ion batteries overcome these drawbacks, with a higher energy density and discharge rate, higher cell voltage, longer cycle life and elimination of “memory” effects [3]. However their major disadvantage is the particular care required when recharging to avoid overheating and permanent damage. Figure 1 shows the relative strengths of the different battery chemistries in terms of their energy and power densities.

Some battery chemistries have problems with shelf life. Standard alkaline batteries have shelf lives of around seven years; while newer lithium based systems (both primary and secondary) have even longer lives. Other secondary (rechargeable) chemistries like Nickel Metal Hydride (NiMH) lose 1 − 2% of their capacity per day of storage.

Secondary batteries provide the option of extending the sensor node's lifetime, relative to that of a primary battery, through their recharging ability. However, this means they need to run in conjunction with another device capable of supplying power. This arrangement is usually desirable as quite often the device supplying the power does so intermittently. A battery can store these bursts of energy and provide the electronics with a stable constant energy interface. A robust system will require electronics to control the charging and discharging of the battery in a way that maximises its life as incorrect charging profiles diminish the battery's usable life.

Two promising new fields of research in battery technology are micro-batteries and flexible batteries. Micro-batteries seek not only to reduce the size of the actual battery but also to improve integration with the electronics they are powering. The goal of micro-batteries is therefore to produce a battery on a chip. The main challenge is overcoming small power outputs due to the surface area limitations of micro-batteries, however work into three-dimensional surfaces seem promising [9]. The second field involves a new breed of lightweight flexible batteries [48] which can be moulded to any shape allowing them to serve a double purpose of acting as structural material, thus reducing the total volume of the sensor node.

### 2.2. Capacitors

Capacitors store energy in the electric field between a pair of oppositely charged conductors. They have significantly higher *power density* than batteries, as they are able to charge and discharge over much shorter periods of time. However, their *energy density* is two to three orders of magnitude lower. This makes capacitors ideal for providing short bursts of high power with low duty cycles giving the capacitor time to recharge before the next burst of power is needed.

This effect may mean a combination of capacitor and battery could solve the power requirement across a normal nodal duty cycle. A battery can be used to provide the low power requirements on sleep and receive mode, while a capacitor can provide the high power required for RF transmission on short duty cycles.

**Figure 2.**
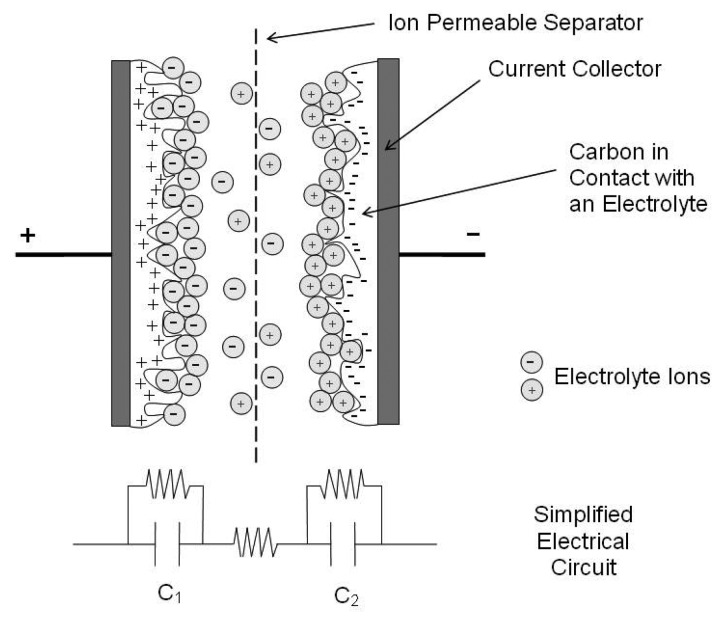
Representation of a charged electrochemical double layer capacitor [23].

Continued research into capacitors strives to increase their energy density, with a new breed of supercapacitors. Figure 2 shows a charged supercapacitor. The critical difference between a supercapacitor and a standard capacitor is in the surface area supplied by the electrode and the thinness of the double layer formed at the electrode-electrolyte interface. In a standard capacitor the area is simply the surface area of a nominally flat plate. However, the use of porous materials such as carbon effectively increases the surface area of each electrode enormously. This allows capacitors with values of the order of 2000 F in packages approximating standard battery sizes.

The simplified circuit shown in Figure 2 hints at a further improvement: Capacitors in series add such that the total capacitance of a cell is given by:

(1) $$\frac{1}{C_{\text{CELL}}}=\frac{1}{C_{1}}+\frac{1}{C_{2}}$$

**Figure 3.**
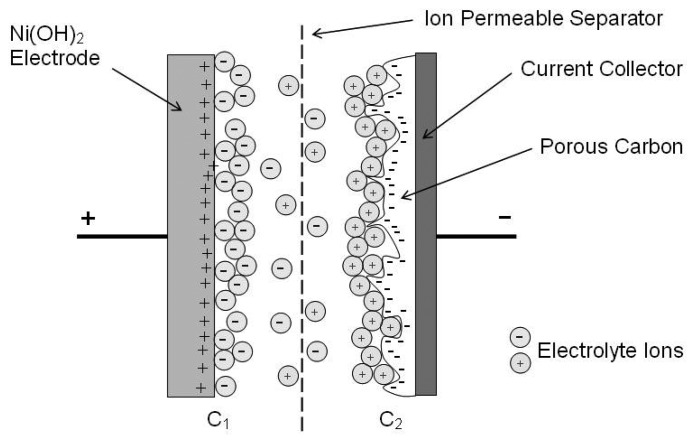
Schematic of an Ni(OH)_2_/NiOOH–porous carbon asymmetric supercapacitor [24].

Thus, for a supercapacitor both C_1_ and C_2_ are large and this leads to a C_CELL_ approximately half the size of C_1_ or C_2_. This has lead to the development of so called asymmetric capacitors, as seen in Figure 3. An asymmetric supercapacitor typically consists of a battery type electrode (usually a faradaic or intercalating metal oxide) and an electrochemical capacitor type electrode (high surface area carbon). In such an arrangement, the carbon electrode has a much greater capacity than the battery electrode. Thus C_CELL_ approaches the capacitance of the carbon electrode alone, resulting in a much larger energy storage capability of a comparable symmetric carbon based supercapacitor. This has lead to development of cells with capacitance values in excess of 8,000 F.

The increase in capacitance values has led to *energy* storage capabilities approaching that of some battery chemistries, such as lead-acid storage cells, and *power* storage capabilities and order of magnitude greater. Critically, the efficiency of capacitors exceeds 90% while batteries have typical values of 60–70%.

There has also been recent work by CSIRO into a combination of lead acid battery and supercapacitor [6]. Although the specific application here is for hybrid cars, the technology should be applicable to sensor nodes. Figure 1 shows a comparison of the energy and power densities of the energy storage devices just discussed: capacitor, supercapacitor and battery.

Some supercapacitors are capable of more than 500,000 charge cycles before noticeable deterioration (compared with about 1,000 for rechargeable batteries) [6]. This factor, along with short charging times and high power densities, make supercapacitors attractive as secondary power sources in place of rechargeable batteries in some wireless sensor network applications [7].

### 2.3. Micro-Fuel Cells

**Figure 4.**
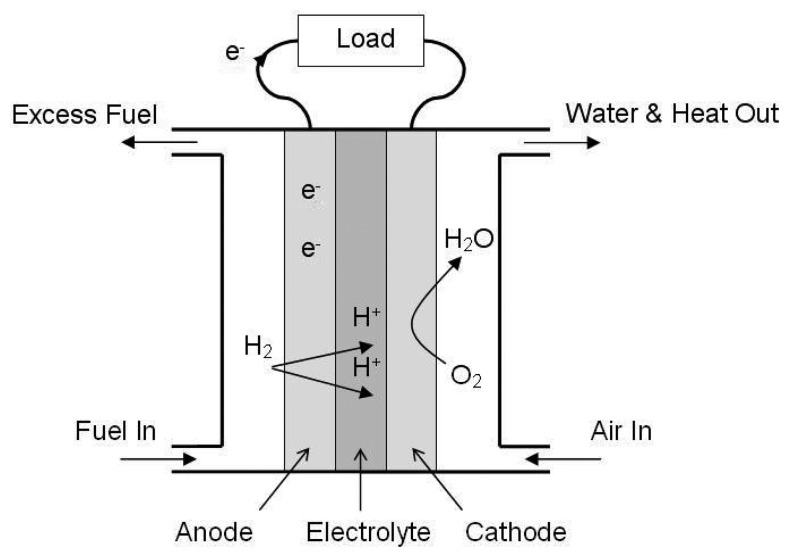
Example of a polymer electrolyte membrane (PEM) fuel cell.

Like batteries, fuel cells convert stored chemical energy into electricity. Generally, liquid fuels have much higher energy density than battery chemistries. In the fuel cell, such as the one shown in Figure 4, a catalyst promotes the separation of the electrons from the protons of hydrogen atoms drawn from the fuel. The electrons are then available for use by an external circuit, while the protons diffuse through an electrolyte to recombine with the electrons and oxygen on the other side producing water molecules [7]. This technology was pioneered for the NASA space program and has been used on large scales for decades but recent work has focused on reducing their size to replace consumer batteries [8].

As with batteries, the major performance restriction of micro-scale fuels cells results from the small electrode surface area. An opportunity may exist to combine the work of Hart *et al.* [9] involving three dimensional surfaces in battery electrodes, with the noted shortcomings of fuel cell electrodes. Another hindrance is the plumbing for the fuel reservoir which at micro-scales is seen as a harder task than micro-fabricating the electrodes. The main issue here is due to flow considerations and ensuring that the fuel flows throughout the cell particularly to the finer tubing at the extremities.

Matsushita Battery has developed a direct methanol fuel cell (DMFC) incorporated with a lithium ion battery. This system is approximately 400 cm^3^, with peak output of 20 W and an average of 13 W [49]. This corresponds to a average power density of 0.03 W/cm^3^. Angstrom Power has completed a six month test program using a hydrogen fuel cell. The fuel is supplied as hydrogen absorbed in a metal hydride. The volume of the fuel storage is around 6 cm^3^, and the fuel cell itself can be made in many forms. The two presently available are a cylindrical, 1 W unit with a volume of 10 cm^3^, and a rectangular 0.38 W unit with a volume of 2.5 cm^3^ [50]. The average power densities for these, including the fuel storage, are 0.06 W/cm^3^ and 0.04 W/cm^3^, respectively.

### 2.4. Radioactive Power Sources

The use of radioactive materials as a power source is attractive due to their extremely high average energy densities, approximately 10^5^ kJ/cm^3^ [10]. Like many other power sources it has been used in the large scale for decades but has not yet fully transferred down to a scale useful for sensor nodes. The main technical reason for this is the lack of a high conversion efficiency mechanism at the micro-scale.

Early research into small scale radioactive energy conversion focussed on thermal heating using the kinetic energy of emitted particles. The heat could be converted into electricity using thermoelectric or thermionic techniques which require high temperatures (300 – 900 K) for efficient operation. This scheme works well for operations requiring power in the Watt to kilowatt range but doesn't scale down for micro-power applications since with reducing size, the surface-to-volume ratio increases, leading to high heat leakage to the surroundings, i.e. thermal heat management at the micro-scale is a tough engineering challenge [11].

**Figure 5.**
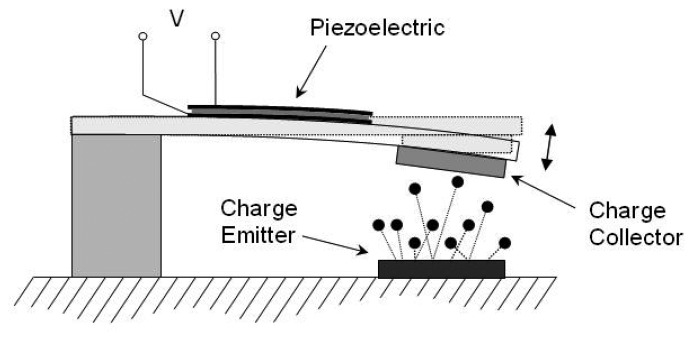
Radioisotope energy harvester.

To date the most promising work for applications in powering wireless sensor nodes is by Lal *et al.* [11] where they have used a radioactive isotope to actuate a conductive cantilever. As shown in Figure 5 the emitted electrons collect on the cantilever which causes an electrostatic attraction forcing the cantilever to bend towards the source. When contact is made the charge differential is dissipated and the cantilever oscillates about its equilibrium position. A piezoelectric plate will convert the mechanical energy of the oscillation into electrical energy. They have demonstrated a power conversion efficiency of 2 − 3% using this radioactive-to-mechanical-to electrical conversion cycle with power outputs in the tens of microwatts, which could power low-power electronics or trickle charge a battery or capacitor.

The weakness of useable radioactive sources is their low power density. Typically, the longer the half-life of an element, the lower the power density. As such they do not by themselves offer a standalone solution to the powering of sensor nodes. However, they are an extremely consistent power source with long lifetimes governed by the half-life of the source which in some cases can be centuries. Because of this they are often put into the energy harvesting category, but strictly speaking they are in fact an energy storage source. Possible uses include extending the life of batteries, charging capacitors, or providing power to applications which need very low power. Due to safety concerns the use of radioactive material is a highly political and controversial topic. As Table 2 shows, although some groups of betavoltaics offer good power density, they also require extensive shielding.

The company BetaVoltaic Industry [12] has indicated that the energy sources shown in Table 2 are probably only suitable for military or space applications and they are investigating other beta sources such as Potassium-40, Molybdenum-100 and Zinc-70. However, other references indicate that Potassium- 40 has substantial gamma radiation (about 11% of all decay events) [14], and Zinc-70 is a stable isotope that does not undergo radioactive decay [14]. Zinc-69 is a beta emitter, but with a half-life of less than 1 h. These factors would seem to preclude these elements from a civilian betavoltaic battery system.

The isotope used in Lal *et al.* [11] is Nickel-63. When a calculation to determine the power capacity of this isotope was completed it revealed power density orders of magnitude less than those quoted in Table 2. It is unclear what the sources of error are. However, the system in Lal *et al.* [11] has been peer reviewed.

It should be noted that there are a number of misconceptions about beta sources of radiation. Although generally less dangerous than gamma emitters, beta sources are still capable of high levels of activity, and in general they should not be handled without adequate protection. The Australian Radiation Protection and Nuclear Safety Authority (ARPANSA) list all of the elements in Table 2, except Cerium-144, Nickel- 63 and Thallium-204 as “Highly Radioactive Sources” if they exceed a defined activity level [15]. This definition is used to determine if a substance needs an *export* permit. All radioactive substances require an *import* permit to Australia.

A second level of hazard comes from the use of lead shielding. Because lead shielding is very effective at slowing down the beta particle it can release a secondary source of radiation. This is similar to X-rays and is called bremsstrahlung, literally *brake radiation*.

In order to reduce the levels of this secondary radiation, it is suggested [16] that materials of low mass number, such as aluminium or plastic, are used as shielding. A shield of 10 mm thick Perspex or 3 mm thick aluminium is suggested. The cited reference gives an example of a 1.7 MeV phosphorus source. That would re-emit 4.6% of its energy as bremsstrahlung with lead shielding but less than 1% with aluminium and less than 0.5% with Perspex. Perspex has the added advantages of being transparent and easier to clean.

## 3. Energy Harvesting

### 3.1. Solar Photovoltaics

**Figure 6.**
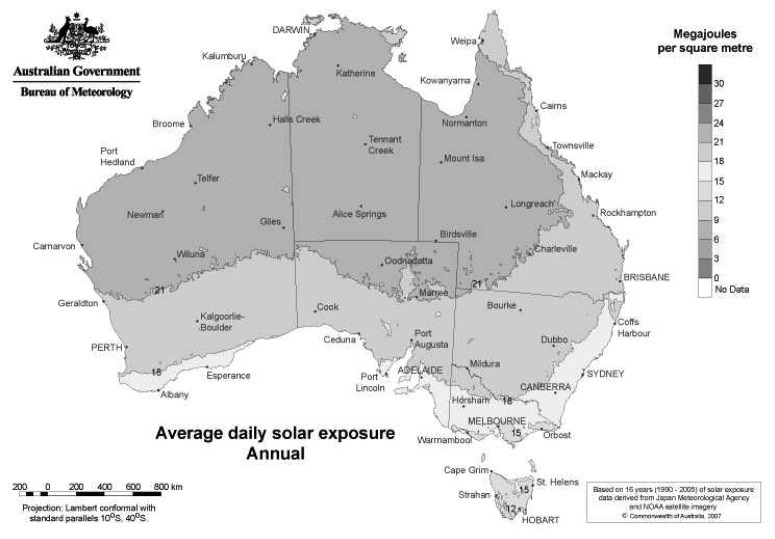
Average daily solar exposure [17].

Figure 6 displays the average daily levels of solar radiation energy falling on a horizontal surface across Australia. It shows that a majority of populated areas receive 15 – 18 MJ/m^2^ (1.5 − 1.8 kJ/cm^2^). There are 3.6 kJ per Wh so this is approximately 0.4 − 0.5 Wh/cm^2^. In terms of daily power there is approximately 0.1 W/cm^2^ peak. This offers a huge potential for wireless sensor node energy scavenging as a solar collector at 12 − 15% efficiency with the area of 25 cm^2^ would produce over 300 mW peak of solar power. This would be more than enough to run most wireless sensor node applications.

There are a number of factors which reduce the power realisable from the high values quoted above. The first and most obvious is that the Sun is only in the sky for half the day, thus the cells will yield no power at night. Therefore some form of secondary storage, such as batteries, will be required in order to use the power throughout the day. Other factors, such as cloud cover and shadowing, block the Sun's rays and drastically reduce the level of incident radiation. In extreme cases the sensor node may be deployed in a location which has no direct sunshine upon it. For example inside an office building the available power levels incident on a solar cell are three orders of magnitude less than outside, directly under the Sun. Commercial solar conversion efficiencies range from a low of approximately 8% to state-of-art values of 20%, with some experimental technologies reaching as high as 35% [18]. Different types of solar converters are more efficient indoors or outdoors due to their spectral responses, thus it is important to know where the node will be deployed when developing a solar harvesting system to achieve maximum results.

The power incident on the collector drops with the cosine of the angle of incidence of the sun's rays i.e. 100% available when the rays are perpendicular to the surface, 87% when they are 30 degrees from perpendicular and none when the light is parallel and thus not directly striking the surface. Large scale solar collectors use solar tracking devices to ensure the cells are always facing towards the sun.

An analysis performed by Thomas *et al.* [18] into the effect of solar tracking on a collector's performance used four different strategies. The first was a standard horizontal flat collector, the next had the collector at a fixed tilt on some optimum angle for the given location, the next had one axis tracking and the last had two axis tracking. Results from the average monthly energies harvested from the four collectors showed that the horizontal flat collector yielded the least energy, the fixed tilt collected 17% more energy, the one axis tracking 50% more and the two axis 54% more energy than the horizontal flat collector. This analysis shows that, as expected, the tracking yields better energy scavenging performance but at the expense of added weight, complexity and cost of the tracking control equipment, so analysis needs to be done to determine the value of adding tracking to a small scale collector used for sensor network applications.

**Figure 7.**
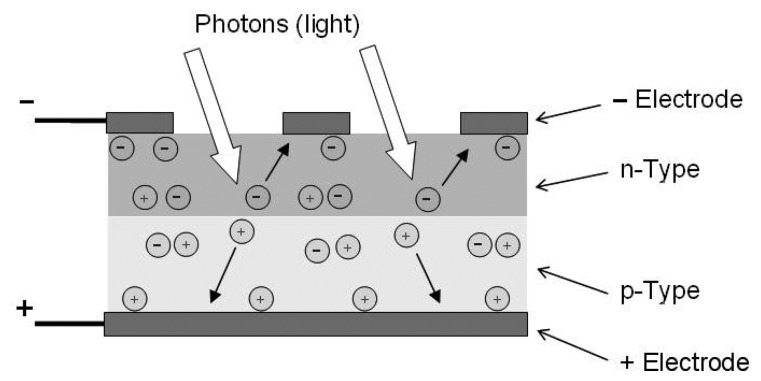
Photovoltaic cell.

The most basic solar converter is a solar cell, which is made of p-n type semi-conductor materials. The p-n materials are positioned such that it forms a p-n diode junction close to the top surface of the solar cell, as shown in Figure 7. When the solar cell is exposed to photon radiation, an electric voltage potential is developed between the p- and n-type materials. A single solar cell has an open circuit voltage of about 0.6 V but can easily be placed in series with other cells to get almost any desired voltage and in parallel with other cells to increase the current.

“Flexible” solar cells are a new technology which may play a role in sensor node applications. This technology has demonstrated efficiencies in the 10−11% range and can be easily integrated as a multifunctional “power skin” in order to provide some mechanical load-carrying capacity, which allows for a reduction in structural mass [3].

**Figure 8.**
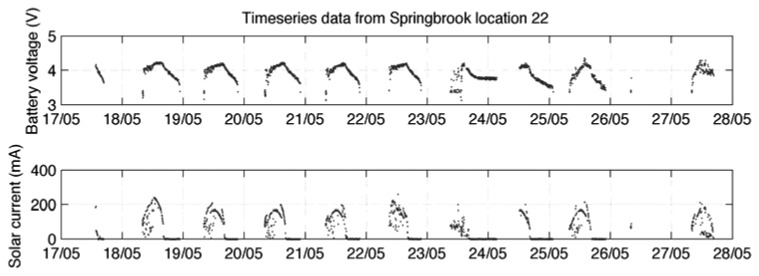
Solar current and battery voltage in full sunlight [25].

**Figure 9.**
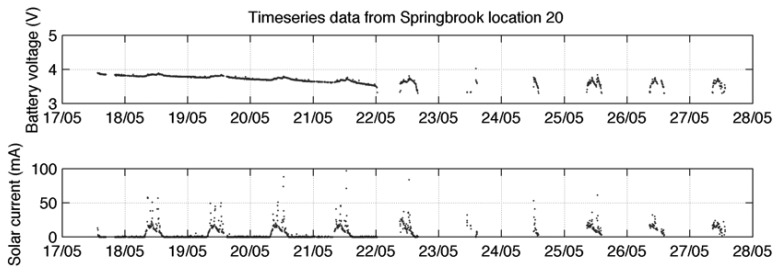
Solar current and battery voltage in partial sunlight [25].

**Figure 10.**
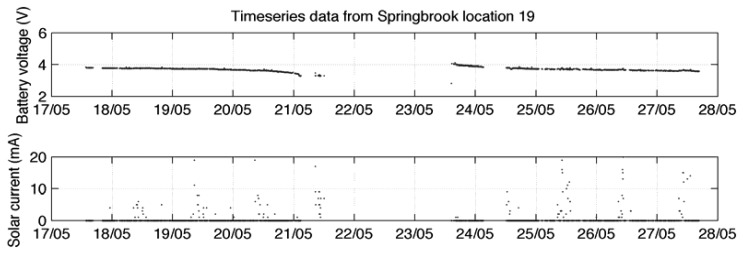
Solar current and battery voltage in low sunlight [25].

Solar cell power is a good resource where direct sunlight is available. However, where there is a deficient solar resource the node may harvest insufficient energy to store excess and only operate during daylight hours. Subsequently, during shorter winter days the node may fail to operate continuously even in daylight as the level of solar energy drops further [25]. Figures 8, 9 and 10 show variations in data reliability based on full sunlight, partial sunlight and low sunlight respectively. Where direct sunlight is available (Figure 8) the solar current peaks between 200 and 400 mA (for the system detailed in [25]). In decreased solar resource areas (Figure 9) this peak occurs between 50 and 100 mA and at very low levels (Figure 10) the peak falls to between at 10 and 20 mA. This is insufficient to keep the battery fully charged and significant data loss occurs.

A number of groups have explored utilising solar power for sensor nodes and have reached the point of offering plug-and-play solar energy harvesting modules. One such system, Heliomote [26], enables energy harvesting, storage, power management while delivering information on solar and battery-state through a basic one wire interface to the node. There are a number of different systems with different design objectives, a summary of which is given in a report by Enviromote [27] which is a system with the objective of increasing its available energy storing capability. These systems incorporate smart electronics to optimise the efficiency of the solar harvester and the battery charging circuit simultaneously. Such electronics are vital in energy harvesting, and this is explored in Section 4.

### 3.2. Thermal Energy

A temperature difference existing between two locations will result in a flow of heat energy from hot to cold in an attempt to develop thermal equilibrium. This heat flow can be exploited to harness useful energy. The process is governed by the laws of thermodynamics therefore its efficiency, the ratio of the useful work extracted out, *W*, to the input heat, *Q*, is constrained by the fundamental Carnot limit. The Carnot efficiency limit applies to all heat engines and generators and can be expressed in terms of the hot, *T_H_*, and cold, *T_C_*, temperatures as,

(2) $$\eta =\frac{W}{Q}=\frac{T_{H}-T_{C}}{T_{H}}$$

This shows that the efficiency is very low for small to modest temperature differences. As an example, a heat source, 5 °C above room temperature (25 °C), could be used to harness energy with an efficiency of 1.6%. Even if that heat source was increased to 100 °C the efficiency would only be 20%. The Carnot efficiency limit is the maximum theoretically possible efficiency; real world conversion devices however do not achieve efficiencies as high as this. Current commercial devices operate below 40% of the Carnot efficiency.

The low efficiencies of this process necessitate a large amount of heat to be transferred in order for a device to harvest a given amount of useful work. The transfer of heat can occur in three different ways; via conduction, convection and/or radiation. Roundy *et al.* [7] derives an analysis to demonstrate the power levels achievable from temperature gradients by assuming heat conduction through a silicon material. At small scales and temperature differentials, convection and radiation are negligible compared to conduction and as such the amount of heat flow can be given by;

(3) $$q=k\frac{\Delta T}{L}$$

where *q* is the heat, *k* is the thermal conductivity of the material, *ΔT* is the temperature difference and *L* is the length of the material.

There are many types of engines designed to extract useful work from sources of heat, examples of which range from thermally powered wrist watches to the engine in your car to nuclear power plants. These engines can be broadly classified into two categories; mechanical and solid state. For the application of harvesting low amounts of power for wireless sensor nodes, from small ambient temperature gradients, solid state devices have the best potential. This is due to their lack of moving parts which facilitates robustness and low maintenance requirements. Life testing of thermoelectric devices has shown their capability for over 100,000 hours of continuous operation [28]. They are compact and light, noiseless in operation, are highly reliable and eliminate power losses in extra conversion steps needed for mechanical engines. The following sections give an overview of the different solid state thermal energy harvesting techniques currently available.

#### 3.2.1. Thermoelectric

Thermoelectricity is by far the dominate solid state conversion technique so a more in depth description will be given for it than the other techniques. It involves the direct conversion of heat into electricity via a phenomenon known as the Seebeck effect. This effect promotes a thermoelectric EMF across two different metals or semiconductors when their junctions are placed across a temperature gradient.

**Figure 11.**
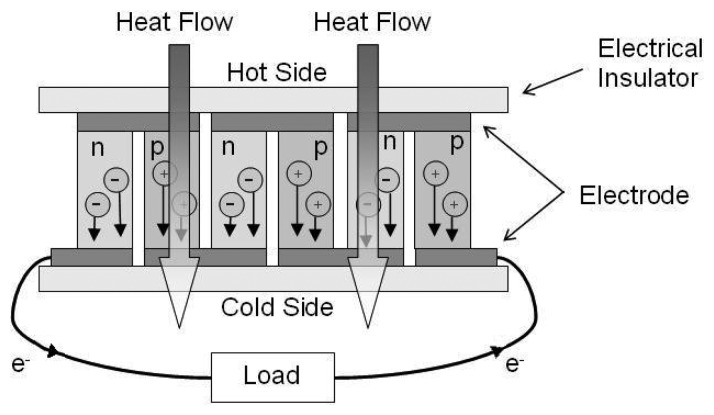
Thermoelectric module.

Modern thermoelectric devices use n- and p-type bismuth telluride semiconductors connected electrically in series and thermally in parallel. They have one end exposed to a heat source and the other to a cooler side in a configuration known as a thermocouple as depicted in Figure 11. The temperature difference across the semiconductors results in a flow of heat which involves the diffusion of charged carriers from the hot to cold side. The charge carriers in the n-type material have a negative charge producing a current from the cold to hot side whereas the carriers in the p-type material have a positive charge producing a current from the hot to cold side, with the total result of an electron current flowing clockwise around the circuit shown.

The voltage produced across a thermoelectric device is proportional to the temperature difference across it and to the difference between the Seebeck coefficients, *S_1_(T)* and *S_2_(T)*, of the two materials. The n- and p-type semiconductors have a negative and positive Seebeck coefficient respectively. As the Seebeck coefficients are functions of temperature, the value of the voltage across a thermocouple exposed to a temperature difference, *T_H_* - *T_L_* , can be found from the integral in Equation 4. This voltage is generally quite small so many thermocouples are usually connected in series to form a thermopile in order to achieve useable voltages. The power harvested by a thermocouple or thermopile is proportional to the square of the voltage and therefore the temperature difference. That is,

(4) $$V=\int_{T_{L}}^{T_{H}}S_{1}(T)-S_{2}(T)dT$$

The efficiencies of thermoelectric generators have remained low and unchanged for the past 50 years. The reason for this is that in order to exploit a temperature gradient the thermoelectric device must be a good electrical conductor to allow the flow of charge but be a thermal insulator to maintain the temperature difference. This is contrary to most conventional materials as good electrical conductors are also good thermal conductors therefore a large portion of energy is transferred across the device as heat and not as electrical energy. The dimensionless thermoelectric figure of merit, ZT, is a measure of this ability and is roughly proportional to the devices efficiency [29]. ZT is given by equation 5, where σ is the electrical conductivity, λ is the thermal conductivity and *S* is the Seebeck coefficient. It has remained around the value of 1 for more than 50 years however modern research into thermoelectric materials is improving this by a factor of over 2 [30].

(5) $$ZT=\frac{\sigma S^{2}}{\lambda }\left(\frac{T_{H}-T_{L}}{2}\right)$$

Improving this *ZT* value is crucial for the widespread implementation of thermoelectric converters as typical commercial converters currently operate at efficiencies of less than 6% [28]. For more information on the current state of the art of thermoelectric power generation, Weiling and Shantung [29], and Riffat and Ma [28] give good summaries on the topic.

**Figure 12.**
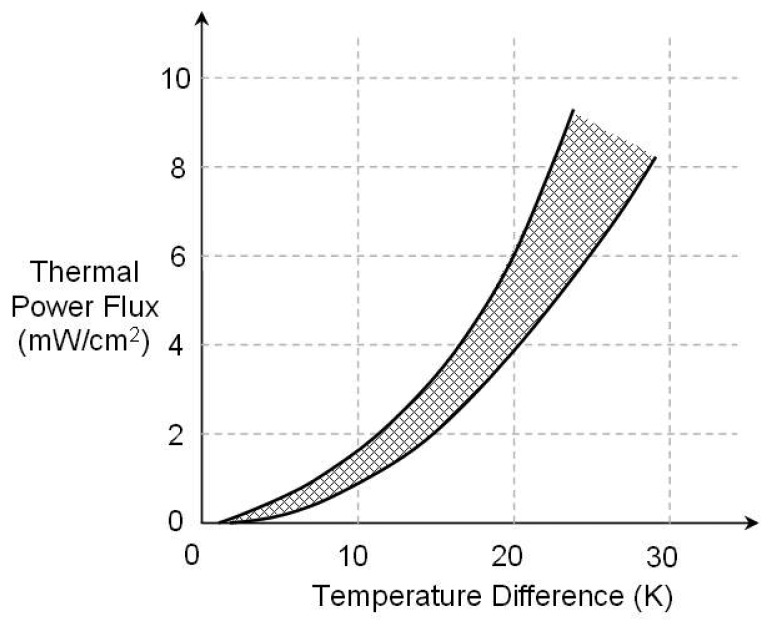
Maximum power flux generated by example Thermoelectric Generators [38].

Ferrari *et al* [38] investigated using a thermoelectric generator to power a wireless sensor node. The paper presents the characterization of three different commercial thermoelectric modules designed for heating/cooling applications. Their analysis included the effects of electrical load resistance, thermal conductivities of the thermoelectric and heat exchanger modules and different temperature gradients. They found that thermoelectrics could be used for their application of powering a wireless sensor node consuming 32 mW, when the temperature difference exceeded 30 K. Figure 12 shows the result of some of their work, displaying the maximum power density generated by the three different thermoelectric generators vs. temperature difference.

#### 3.2.2. Thermionic and Thermotunnelling

Another range of solid state heat engines which have been around for decades are those based on thermionic conversion. A simplified description of a thermionic converter is a system where electrons are ejected via thermionic emission from a hot electrode over a potential barrier to a cooler electrode. The barrier the electrons must overcome is known as the work function of the material and is essentially the heat of vaporization of the electrons from the surface. Due to this, the thermionic conversion works best with large temperatures. Although thermionic conversion has better efficiencies than thermoelectric devices, its reliance on high temperatures would make it unsuitable for most wireless sensor applications.

A method similar to thermionic is thermotunnelling, which narrows the potential barrier using properties of quantum physics known as quantum tunnelling. This technology seems plausible to use for small temperature gradients for lower power applications [31].

#### 3.2.3. Heat Sources

Section 3.1 outlined the enormous potential for solar energy harvesting. Rather than use the conventional conversion method of photovoltaics, a growing field is exploring converting solar energy indirectly through thermal energy harvesting. The advantage of switching to thermal harvesting lies in its cheaper cost than the expensive photovoltaic cells plus results point towards the capability of better efficiency. For large scale operation, arrays of mirrors are used to reflect and concentrate the Sun's power to a single point where the temperature rises to hundreds of degrees Celsius. On a smaller scale suitable for wireless sensor nodes, a black surface facing the Sun will absorb the Sun's rays and heat up relative to its shaded underside. By sandwiching a thermoelectric or other thermal energy harvester between the two sides the difference in temperature of the top and bottom faces can be used to generate power.

**Figure 13.**
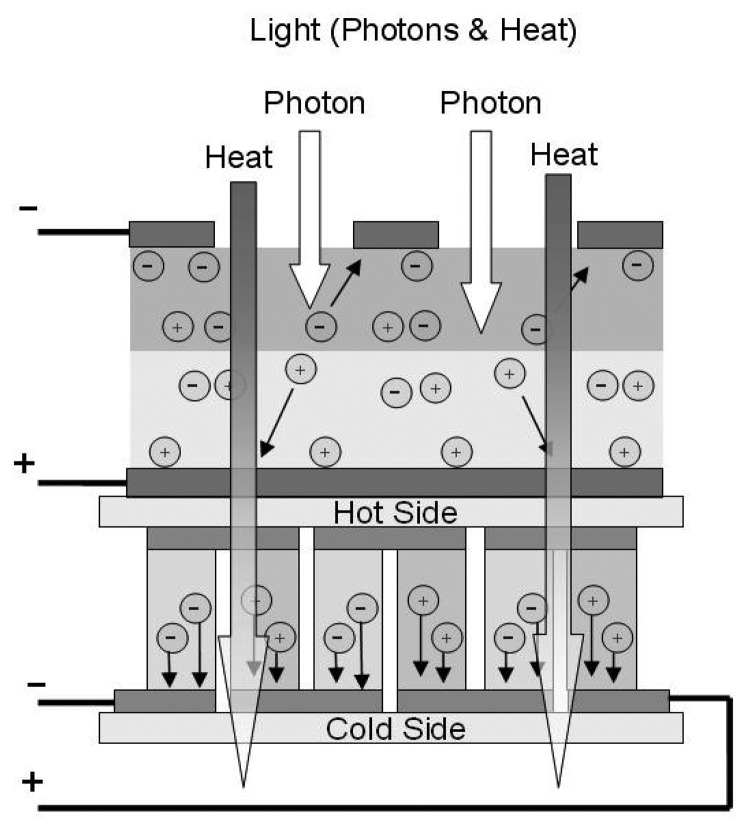
Hybrid Solar PV/thermoelectric harvester.

Yu *et al.* [32] investigated the use of a hybrid power system for wireless sensors which incorporated solar and thermoelectric conversion. Solar cells heat up when in operation, so to harness this waste heat they attached thermoelectric harvesters underneath the cells with a heat sink underneath the thermoelectric harvesters to the atmosphere, as can be seen in Figure 13. In their experiments, for a solar irradiance of 744 W/m^2^ and ambient temperature of 34 °C, they found that the rear of the solar cells reached 61 °C. In other research by Wang, [33] temperatures measured at the rear of solar cells reached over 70 °C in stronger summer light. The advantages of harvesting this relatively large 30-40 K temperature difference are twofold. Firstly and most obviously the thermoelectric devices are harvesting and providing extra power to the sensor node. The second benefit is that it increases the efficiency of the solar cell above its efficiency without the thermoelectric device there. This is due to the fact that a solar cell's efficiency drops with increasing temperature by about 0.4% per degree. By including the thermoelectric device to harness heat energy from the cell its temperature drops and efficiency increases. In their experiment Yu *et al.* found that the rear of the cells equipped with thermoelectric harvesters were 13 K colder than those without and measured a 5.2% increase in their efficiency.

**Figure 14.**
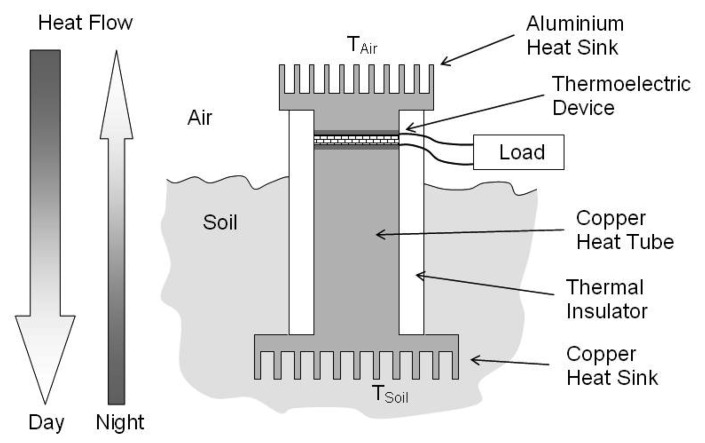
Simplified Diagram of Temperature harvesting device.

Another potential source of heat energy is the soil to air boundary. Air temperature will rise during the day in response to the Sun's radiation and then cool during the night. These temperature changes will be transferred to the ground although phase shifted and attenuated with depth. Because of this there will exist a temperature difference between the air and the ground which is generally positive during the day and negative at night. Figure 14 illustrates this concept where air side heat exchanger thermalises with the ambient air, likewise the heat exchanger in the soil thermalises with the ground at a desired depth. The heat pipe then transfers this heat to/from the thermoelectric device allowing the temperature difference from across tens of centimetres of soil to be applied directly across the much thinner TEMG.

Stevens [34] showed that the magnitude of the available temperature difference attenuates with depth and that there is also a depth dependent phase shift between the air and soil temperatures as they fluctuate throughout the day and night. As a consequence there is an optimal depth for the ground side heat exchangers for maximum temperature difference and therefore power harvesting. By placing the heat exchangers at this optimum depth a 7% increase in the temperature difference over the difference that could be achieved by placing the heat exchangers at an infinite depth.

Lawrence [35] investigated the effects of the thermal conductance of heat sinks. In his experimental set up he did not actually measure the power harvested but indirectly predicted an average value of about 50 μW. This low power value lead him to claim that the results do not appear promising and that according to their temperature data most of the temperature drop in the system was across the heat pipe, up to 4.5 K out of the 10 K total across the air and soil. Meydbray *et al.* [36] experimented on the effects of the thermoelectric generator module surface area when exploiting the soil to ambient air temperature difference. They ran 3 modules with different surface areas for 110 hours with the results shown in Table 3, indicating a strong dependence on the surface area. Additionally during the course of this experiment their data also showed a large decrease in power output during times of cloud cover. These results mirror the sub-milliwatt power values obtained from Lawrence's experiments.

Other potential areas of thermal difference suitable for thermoelectric devices include industrial machinery, mammalian bodies and civil structures. For the application of wireless sensor nodes deployed around industrial machines, piping or vents, there is an abundant source of otherwise wasted thermal energy available. This source is very site specific but for sensors developed to monitor such equipment it is logical to power them from the rich energy source in its environment. An example of this is the automobile industries use of sensors in the engine to provide control units with information, with the sensors being powered by the waste heat from the engine. Bodensohn *et al.* [37] report thermoelectric generators supplying autonomous sensor systems with up to 7 mW of power utilizing this heat source.

The human body self regulates its temperature at a constant 37 °C. Harnessing this against the ambient air temperature offers a source for thermal harvesting for sensor nodes applied on the human body. Many companies producing body worn products have already developed devices which utilise the small temperature difference between our body heat and the ambient temperature, generating power on the order of microwatts demonstrating that similar techniques can be applied to sensor nodes [4]. An example of this is Seiko Thermic watch that uses the small thermal gradient between the wearer's arm and the ambient air to generate microwatts of power to run the movement of its mechanical clock.

Meydbray *et al.* [36] investigated exploiting the temperature difference between solid structures and the ambient air. They reported an average power density on the order of 5 mW/m^2^ from their air-solid structure setup.

### 3.3. Mechanical

The final collection of energy harvesting technologies falls under the umbrella of mechanical energy harvesting. Mechanical energy harvesting involves converting the ambient movement or kinetic energy in the devices environment into electric energy. The final technology, vibration harvesting, includes an in depth look into the power conditioning necessary to transition from the raw energy outputted from the harvester to an interface useable by the node. Note that parallel techniques need to be considered for all energy harvesting technologies but, for the sake of brevity, have only been outlined in depth for the one technology in this review.

#### 3.3.1. Fluid Flow

The flow of any fluid (gas or liquid) can be converted to electricity with a variety of techniques. At the macro scale, the use of wind turbines is becoming more common. However, at the scale needed for sensor networks, more novel approaches are required, due to viscosity effects.

The potential power from a moving fluid is given by:

(6) $$p=\frac{1}{2}\rho Av^{3}$$

where *ρ* is the density of the fluid, which for air is 1.2×10^−6^ kg/cm^3^, and for water is 1000×10^−6^ kg/cm^3^, *A* is the cross-sectional area the fluid is flowing through, which in the large scale is the area swept by the rotors, and *v* is the velocity of the fluid.

Betz's Law, analogous to Carnot efficiency, is a measure of the maximum efficiency of a wind turbine. This law determines the maximum efficiency of a turbine to be approximately 59%. Large scale wind turbines operate at maximum efficiencies of about 39%. Efficiency is dependent on wind velocity and average efficiencies are usually around 20% [7].

There is a quite reasonable potential from wind power, however, only in specific applications where the sensor node would be deployed in locations subjected to a constant wind resource would pursuing this powering option be attractive. Also, it is undesirable to have a system which is designed to be left unattended for long durations, with so many moving parts, some of which are exposed to the elements, making reliability an issue.

It should also be noted that typical wind power maps are often quoted at altitudes of 80 meters, which is a typical hub height of a large wind turbine. Sensor networks would typically be deployed closer to the ground, and due to boundary layer effects, the energy available from air flow will be much less than that available at 80 meters.

The process of capturing energy from water is similar to that for air movement. The dependency of the maximum power on the density of the fluid means that for any given volume moving at a fixed speed there is approximately 3 orders of magnitude more power available in water.

Of course any fluid moving can be harnessed. For example an oil pipeline may be a source of fluid energy. The problem of fouling is still a problem with liquid based fluids and consideration of the environment is required before deployment of any sensor network.

Some novel methods of generating power from fluid flow have been researched. A number of systems for use in both water and air have been proposed based around the fluid causing a piezoelectric fin or ‘eel’ to oscillate due to vortex shedding (see for example reference [19]). Conversion of this captured piezoelectric energy to useable electrical energy is complex and addressed in Section 4.

As a final example of air flow scavenging, mine vent bags are used to provide large amounts of fresh air to underground mining operations. The power supplies for these systems are large arrays of fans with typical power consumption of 100 kW across the array. Typical vent bag diameters of 1 m would give air speeds well in excess of 20 m/s and power outputs in the order of 1 W/cm^2^. Conversion of this rotational energy to electrical energy suitable for battery storage is a relatively simple process involving rotational electromagnetic generators.

#### 3.3.2. Pressure Variations

Another energy harvesting technique for which no research has been found on the small scale, is exploiting pressure variations. Roundy *et al.* [7], however, gives a simplistic theoretical analysis of the potential of this resource, assuming 100% conversion efficiency [7]. For a fixed volume *V* of gas, the change in energy *ΔE* due to a change in pressure *ΔP* is:

(7) ΔE=ΔPV

In order to calculate a reasonable daily pressure change there are two approaches available. The first is a direct calculation using the daily change in pressure. The second is via the ideal gas equation with inputs from the daily temperature variation.

Although local pressure can change by as much as 30−40 mbar (3−4 kPa) during cyclonic weather events, a more typical daily pressure change is 3 mbar (300 Pa) [20]. With a device volume of 1 cm^3^ this will provide 300 mJ/cm^3^. With two pressure cycles in one 24 h period, this will provide 7 nW/cm^3^. This assumes 100% efficiency and offers no clue as to the mechanism that could be utilised to capture this energy.

A second source of energy available is via the mechanism of a daily temperature variation. A typical variation over a 24 h period might be 10 K. Using the ideal gas equation;

(8) ΔPV=nRΔT

Where assuming a fixed volume *V* of 1 cm^3^, n = 40.8 × 10^−6^ mol of helium gas, *R* = 8.3 J/K/mol and *ΔT* =10 K this temperature change would result in a useable volumetric energy of 3.4 mJ/cm3. With an assumption of one cycle per day, this corresponds to a power density of 39 nW/cm^3^. As the amount of energy is dependant on the number of moles of the gas that is available per cm^3^, this may be increased by pressurising the vessel.

In order to achieve 1 μW/cm^3^ from a system such as this (assuming 100% efficiency), approximately 0.0005 mol/cm^3^ of the working fluid would need to be compressed into the mechanism. This is approximately 12 bar of pressure. The mechanism to extract energy from such a system remains speculative.

#### 3.3.3. Vibrations

A field which has gained a lot of attention for its potential in micro-scale power generation is vibration energy harvesting. Its attractiveness compared to that of other energy harvesting generation methods is the availability of vibrations in most environments and the device's ability to function inside other structures. For example, a vibration harvester can be implanted in a human body for medical monitoring or be deployed inside a concrete wall during its construction for lifetime structural monitoring.

Vibration harvesting involves the conversion of the kinetic energy inherent in mechanical vibrations into electricity. There are three known mechanisms by which this can be achieved: electromagnetic, electrostatic and piezoelectric conversion.

Electromagnetic conversion utilises Faraday's law to induce an electric current in a coil of wire when it is subjected to a changing magnetic flux. By coupling either the coil or a magnet to the vibrations and having the other mounted in an inertial mechanical frame, relative motion is induced between the two. This results in a changing magnetic flux through the coil and thus power generation. On the micro-scale the AC voltage outputs are too low to allow rectification, rendering it useless at this time.

Electrostatic conversion involves designing a system whereby the external vibrations change the distance between two charged capacitive plates held at constant voltage (*V*) or constant charge (*Q*). As the distance (d) between the plates changes their capacitance (*C*) also changes and thus the energy (*E*) can be harvested as:

(9) $$E=\frac{1}{2}QV^{2}=\frac{Q^{2}}{2C}$$

where *C*=*ε_0_Lw*/*d*, and ε_0_ is the dielectric constant of free space, *L* is the length of the plates, *w* is the width and *d* is the distance between the plates.

This method has the advantage of being easily integrated into micro-systems with silicon micromachining and being able to increase energy density with applied voltage, however it does require a separate voltage source to “kick-start” it, and due to its complexity does have some practical difficulties [21].

When a mechanical stress is applied to a piezoelectric material, a charge separation is induced across the material. At the moment the most simple and effective way developed to utilise this phenomenon in vibration harvesting is by mounting the piezoelectric material on a cantilever beam with a mass weighted at its end. This beam oscillates when subjected to vibrations, inducing mechanical stress on the piezoelectric material allowing it to harness power by damping out the oscillation. This method is more difficult to integrate into micro-systems than electrostatic conversion but does not endure the same difficulties the previous method has.

Thus both conversion techniques show promise for vibration harvesting and are the subject of many research efforts around the world. The harvesters developed, range from 1 – 75 cm^3^, exploit vibrations from 50 Hz to 50 kHz, induce oscillations between 0.5 μm to over 1 mm, and produce powers that range from tens of μW to over a mW [4].A recent analysis indicated that vibrational micro-generators (order of 1 cm^3^ in volume) may have power densities of up to 4 μW/cm^3^ peak from typical human motion (5 mm motion at 1 Hz). Machine induced stimuli (2 mm motion at 2.5 kHz) may have a power density of up to 800 μW/cm^3^ peak [22]. The results shown possible by this analysis surpass existing devices' actual performance by one to three orders of magnitude, suggesting that researchers have plenty of room for improvement in this field.

Most devices designed to harvest this energy utilise the oscillation of a proof mass resonantly tuned to the environment's dominant vibrational frequency. This resonance condition is vital for efficient conversion. As vibrations in different environments come in a wide range of frequencies and amplitudes which are often stochastic in nature, a robust system needs to be able to dynamically tune its parameters to match the varying peak in the input vibration's frequency spectrum. So far most work has focussed on systems subjected to vibrations of single static frequencies and hence have not addressed the need for dynamic tuning.

## 4. Power Conditioning Considerations

None of the energy harvesting techniques outlined in Section 3 produces stable DC power readily useable by the node. As such each technology needs to employ a power conditioning strategy to transition from the raw energy outputted from the harvester to an interface useable by the node. The following section outlines power conditioning aspects for the particular case of vibration energy harvesting. For the sake of brevity only vibration energy harvesting will be considered, as the reported techniques can be easily carried over to other energy harvesting technologies.

The electrical energy created by the vibration energy harvester needs to be conditioned before being used or stored for later use by the sensor node. One method for conditioning the electrical energy is to use a passive rectifier. Passive rectifiers consist of a diode bridge and a large filter capacitor [39] to [41]. The diode bridge provides the same polarity of output voltage for any polarity of the input voltage. The filter capacitor helps smooth the output voltage from the load.

**Figure 15.**
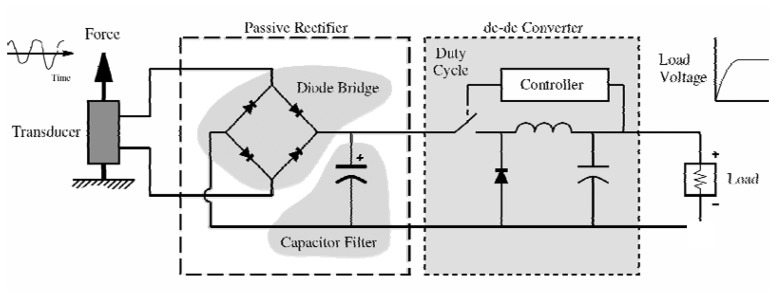
Passive rectifier with dc-dc converter circuit.

In order to improve the harvesting efficiency of this rectifier technique, researchers have added a dc-dc converter [40], as shown in Figure 15. By controlling the switching duty cycle of the dc-dc converter via a control algorithm Ottman *et al.* [40] found harvesting efficiencies (i.e. mechanical-to-electrical conversion) could be greatly increased. Although, Ottman *et al.* report a 325% increase in harvested power over the passive diode bridge technique for periodic disturbances, their results are not a harvesting efficiency, rather they are the improvement across the dc-dc converter.

Under laboratory conditions the passive rectifier dc-dc converter technique looks encouraging as it attempts to crudely consider the dynamics of electromechanical (or vibration energy harvesting) systems, and adapt to slow changing disturbances (less than 1 Hz). However, the technique fails when vibration disturbance are quasi periodic and/or broadband.

Vibration sources are often assumed to be periodic which also limits the applicability of present vibration energy harvesting techniques. Not only are most ‘real-life’ vibration sources broadband, the dynamics of the electromechanical system (mechanical structure, transducer, rectifier circuitry and load) also vary with changing operating and environmental conditions, such as temperature, which can affect the harvesting performance [42] and [43]. Additionally, harvesting performance is also affected by transducer nonlinearities, in particular electroactive polymer materials such as Polyvinylidene Fluoride (PVDF).

Despite the net generation of electrical energy from a transducer with a passive rectifier dc-dc converter technique [39] and [41], the transducer still needs to overcome the semiconductor diode voltage drop of the rectifying/conditioning circuit before vibration energy can be harvested. This voltage drop is typically 0.6 volts and therefore the transducer needs to generate a voltage greater than 0.6 V before an electrical current can be harvested or flow into the load. This is critical when vibration energy harvesting systems utilize a transducer with low internal impedance such as electromagnetic transducers.

In an attempt to overcome diode voltage drop and broadband issues for shunt vibration control, Fleming *et al.* [42] developed a novel active rectifier to simulate shunt impedances. The technique was initially proposed as a self-powering shunt impedance, as shunt vibration controls are required to simulate large reactive impedances, of the order of kHz. Fleming's *et al.* self-powering shunt demonstrated that it could simulate impedances and provide vibration control to a mechanical structure, however, it was unable to power itself. It appeared the self-powering shunt controller could not be autonomous due to the highly reactive electrical power flowing around the circuitry for shunt impedances. Despite the lack of autonomy, the potential to increase vibration energy harvesting efficiencies was realised.

Scruggs [44] also theoretically considers using an active rectifier, similar to that of Fleming *et al.* [42]. Scruggs found that by controlling the voltage and current, or impedance, for the active rectifier the vibration energy harvesting system could be reduced to a standard optimal control problem, which may be solved to maximise the harvesting efficiency performance. This system works well, however, in order to compensate for discrepancies in the model, operating and environmental changes in the system, the controller is ‘de-tuned’, and hence reduces the effective harvesting efficiency performance.

In order to address the changing operating and environmental conditions, as well as the broadband issues, a novel adaptive vibration energy harvesting technique was proposed by Ward *et al.* [45]. The technique demonstrated the use of an adaptive learning algorithm to maximise the amount of vibration energy available i.e. increase the energy harvesting performance. Results of an experimental apparatus using an electromagnetic transducer showed harvesting (mechanical-to-electrical energy conversion) efficiencies of 27 − 34%.

While most researchers have been concerned with rectifier circuit efficiency, some researchers have attempted to investigate the harvesting efficiency i.e. mechanical-to-electrical energy conversion [45] and [47]. Ultimately the cost, size, weight and energy output of vibration energy harvesting will be important for wireless sensors nodes.

## 5. Discussion

This review has identified the developing problem inherent with present energy and power supply methods for wireless sensor nodes. One issue is modern battery energy densities failing to improve at the same rate as other components in a wireless sensor node. This leads to existing sensor node volumes being dominated by that of the battery volume. In addition, the power demands of sensor nodes can range up to 6 orders of magnitude. This range is caused by the varying power demands of different modes, such as transmission, data collection or reception of signals. To further complicate the issue, these modes are done varying duty cycles. Recent solutions involve catering for the largest demand leaving the power system massively over sized for large portions of the node's operating time.

Reviewing the work into power technologies for sensor nodes reveals that there are many different approaches which seem to have the capability of solving the aforementioned problems. Energy harvesting delivers long life, while micro-batteries provide back-up power and micro-supercapacitors enable high power pulses. However, to date there is no ‘one size fits all’, robust system available on the marketplace to provide power for wireless sensor networks for an arbitrary application. One reason for this is due to the bulk of research focusing on one particular technology only for the solution. The problem with this is that in scenarios where one technology may thrive, others may fail, while in a different scenario the first technology may fail while others thrive. This has the effect of making these power systems very application specific. This seems to call for a power system which utilises a hybrid solution of many different technologies able to cater to any scenario. In the last few years this idea has been investigation with papers starting exploration of this scheme [51] to [54].

Research thus far into hybrid power solutions for wireless sensor nodes has been elementary, mostly outlining the potentials and benefits of such systems but failing to get into the detail of how such systems would be optimised. The problems faced in the integration of individual energy storage and harvesting technologies for varying power loads is similar to that of faced by distribute energy or micro-grids. Just as a micro-grid relies on smart software and electronics to integrate numerous sources and loads, to control and manage energy and power, and to provide system stability, so to should the sensor node's “nanogrid” rely on such smart aids to ensure its optimal operation.

Short of a ‘one size fits all’ solution for powering sensor nodes, designers need to choose the right mix of energy storage and harvesting options for their particular application. Once again, drawing inspiration from micro-grids reveals the strength of the National Renewable Energy Laboratory's (NREL) HOMER™ software. HOMER™ is considered to be one of the world's most powerful and widely used micro-power design models. HOMER™ simulates and optimises stand-alone and grid-connected power systems comprising any combination of wind turbines, photovoltaic (PV) arrays, run-of-river hydro power, biomass power, internal combustion engine generators, micro-turbines, fuel cells, batteries, and hydrogen storage, serving both electrical and thermal loads [55]. A similar software package for use by sensor network “nanogrid” designers would prove beneficial.

Some of the individual technologies themselves showed areas of achievable improvements. As mentioned in the vibration harvesting section work into dynamic resonant tuning of the oscillating harvester is lacking as well as a harvester with more than one degree of freedom. Also there seems to be some synergy between the harvesting techniques employed for vibrations and the radioactive actuated piezoelectric cantilever, perhaps a device could be made incorporating both giving the advantage of the consistent power supply of the radioisotope to balance out the intermittent, stochastic nature of the vibration source, and the advantage of high power density of vibration harvesters to balance out the low power density of the radioisotope.

The use of flexible batteries and flexible solar cells seems beneficial due to their ability to double as structural components for sensor networks. This factor may allow them to be used as part of the node structure and thus cut down on volume and mass of the node. The use of temperature differentials was discussed; unfortunately however the low efficiencies that come with environmental differentials of approximately 10 K may limit the use of this source of energy except for specific applications near heat sources. Finally, two energy harvesting techniques were found for which no research could be uncovered at the micro-scale. These were wind power and pressure variations. At the micro-scale these may offer some interesting areas of study. Due to the scaling issues the utilisation of these forms of energy at this scale may require altogether different technologies.

Table 4 is a summary of technologies discussed in this review. Generally, all technologies will require secondary storage except batteries, capacitors and fuel cells. Capacitors are similar to rechargeable batteries, although with differing volumetric power and energy measures. Fuel cells are similar to primary batteries, except for the use of a liquid fuel rather than a chemical paste.

Commercial availability is a determination of whether a member of the non-scientific community can buy the product. All of the noted technologies, except those involving pressure gradients, are available at an academic level. Finally, it should be noted that most technologies have a very wide range of available performance figures. This is a reflection of the wide range of methodologies that exist even within the same general theme. As technologies mature, these levels of variance should be expected to narrow.

## 6. Conclusion

The information presented in this paper is provided as a review of the present state-of-the art technology in powering wireless sensor nodes. These nodes are, by their nature, small in size and require a correspondingly small source of power.

Many of the technologies presented are based on devices that work at the macro scale. However, when scaled down to nodal sizes, their efficiencies become prohibitively small. Of the systems that exist at the moment, the best source of energy for short term use (less than one year) remains the battery. Fuel cells are quickly growing in capacity and it should be expected that they will equal and surpass battery storage capability within two years. While interesting, the political and practical aspects of betavoltaics would seem to preclude their widespread use in civilian application.

In terms of energy harvesting, the outstanding contender at the moment is photovoltaics. Vibration harvesting offers some opportunity where size or location disallow solar. The practical applications behind pressure and temperature change methods are presently not mature enough, though the science appears well understood.

## Figures and Tables

**Table 1.** Consumption parameters for some example wireless sensor nodes [1].

**Node Name**	**Sleep Mode (μA)**	**Transmit Mode (mA)**	**Receive Mode (mA)**	**Duty Cycle (mA)**	**Operating Voltage (V)**	**Batteries Required**	**Battery Life (Days)**
Fleck3	80	36.8	18.4	0.27	3.3	3	440
XBee™	10	45	50	0.51	2.8	2	230
MICAz™				0.70	2.7	2	170

**Table 2.** Decay sources (information based on [12] and [13]).

**Element**	**Power Capacity (W/mole)**	**Power Capacity (W/gm)**	**Half Life (yrs)**	**Lead Shielding (mm/W)**
Caesium-137	20	0.15	30.2	80
Cerium-144	3600	25	0.78	150
Nickel-63	0.42	0.0067	100	<5
Promethium-147	50	0.34	2.6	<1
Strontium-90	80	0.88	28	70
Thulium-170	2200	12.9	0.35	30
Thallium-204	160	0.78	3.8	30

**Table 3.** Output from thermoelectric modules using an air-soil thermal gradient [36].

**Module**	**Power Total (mWh)**	**Power Average (mW)**	**Area (cm^2^)**
1	2.5	0.0228	9
2	7.8	0.071	33
3	63.3	0.575	131

**Table 4.** A summary of technologies (based on [7] with data from [4-5, 9, 11] and [17]).

**Power Source**	**Volumetric Power (μW/cm^3^)**	**Volumetric Energy (J/cm^3)^**	**Secondary Storage Required**	**Commercially Available**
Battery – Primary		1,200 – 3,800	No	Yes
- Secondary		600 – 1,100	N/A	Yes
Super Capacitors		10 - 20	N/A	Yes
Micro-Fuel Cell ^1^		1,000 – 3,000	No	No
Betavoltaics	0.1 - 0.6	1,000 – 2,000	Yes	No
Solar ^2^ – Outside	15,000	1,000 – 2,000	Yes	Yes
- Inside	10	0.8 – 1.2	Yes	Yes
Temperature	5 – 100,000		Yes	No
Gradient ^3^				
Fluid Flow - Air -	200 – 800		Yes	No
Water	500,000		Yes	No
Pressure Gradient	< 10	0.02 - 0.05	Yes	No
Vibrations	4 ^4^ – 800 ^5^		Yes	Yes^6^

1. Based on a methanol fuel cell at 5% efficiency.

2. Normally specified as per cm^2^ rather and cm^3^.

3. Power output is very dependant on magnitude of the temperature change. Normal atmospheric temperature fluctuations give very low power outputs.

4. Human based motion, 5 mm at 1 Hz.

5. Machine based motion, 2 mm at 2.5 kHz.

6. Systems based on periodic motion are available, no known systems based on random motion.
